# Exploring spiking neural networks: a comprehensive analysis of mathematical models and applications

**DOI:** 10.3389/fncom.2023.1215824

**Published:** 2023-08-24

**Authors:** Sanaullah, Shamini Koravuna, Ulrich Rückert, Thorsten Jungeblut

**Affiliations:** ^1^Industrial the Internet of Things, Department of Engineering and Mathematics, Bielefeld University of Applied Sciences and Arts, Bielefeld, Germany; ^2^AG Kognitronik & Sensorik, Technical Faculty, Universität Bielefeld, Bielefeld, Germany

**Keywords:** spiking neural networks, neuron behavior, performance comparison, classification tasks, biological plausibility, computational model, neural network

## Abstract

This article presents a comprehensive analysis of spiking neural networks (SNNs) and their mathematical models for simulating the behavior of neurons through the generation of spikes. The study explores various models, including *LIF* and *NLIF*, for constructing SNNs and investigates their potential applications in different domains. However, implementation poses several challenges, including identifying the most appropriate model for classification tasks that demand high accuracy and low-performance loss. To address this issue, this research study compares the performance, behavior, and spike generation of multiple SNN models using consistent inputs and neurons. The findings of the study provide valuable insights into the benefits and challenges of SNNs and their models, emphasizing the significance of comparing multiple models to identify the most effective one. Moreover, the study quantifies the number of spiking operations required by each model to process the same inputs and produce equivalent outputs, enabling a thorough assessment of computational efficiency. The findings provide valuable insights into the benefits and limitations of SNNs and their models. The research underscores the significance of comparing different models to make informed decisions in practical applications. Additionally, the results reveal essential variations in biological plausibility and computational efficiency among the models, further emphasizing the importance of selecting the most suitable model for a given task. Overall, this study contributes to a deeper understanding of SNNs and offers practical guidelines for using their potential in real-world scenarios.

## 1. Introduction

Artificial General Intelligence (AGI) strives to emulate human-like intelligence in machines, encompassing the ability to perform a wide array of cognitive tasks with high precision, robustness, and efficiency (Goertzel, 2014). As the pursuit of AGI continues, researchers have been exploring innovative brain-inspired approaches to achieve these ambitious goals. In this context, several representative studies have emerged, showcasing promising advancements in brain-inspired intelligence and their potential to outperform state-of-the-art artificial intelligence systems (Mehonic et al., 2020). These groundbreaking works have garnered significant attention in the field, as they lay the foundation for high-level intelligence, accuracy, robustness, and energy efficiency. Therefore, some representative studies exemplify the state-of-the-art efforts in brain-inspired artificial intelligence, offering valuable and inspiring new directions for achieving AGI with unprecedented capabilities (Stimberg et al., 2019; Sanaullah et al., 2022a,b). By combining the principles of neuroscience with advanced machine learning techniques, such as SNNs, these novel approaches hold the potential to revolutionize the field and drive AGI toward realization.

SNNs are a type of neural network model that has gained considerable attention in recent years due to their ability to simulate the behavior of biological neurons in the brain (Ghosh-Dastidar and Adeli, 2009b; Sanaullah et al., 2023a). SNNs are characterized by their discrete time steps, where the neurons generate spikes when the input reaches a certain threshold (Tavanaei et al., 2019; Sanaullah et al., 2020). This is similar to how biological neurons work, where they communicate with each other through the generation of action potentials or spikes (Tavanaei et al., 2019).

To build an SNN, a mathematical model of the spiking behavior of neurons is required. Several models have been developed, each with its own advantages and limitations. One of the most commonly used models is the leaky integrate-and-fire (*LIF*) model (Brunel and Van Rossum, 2007), which models the behavior of neurons as a leaky capacitor that charges and discharges over time. The Adaptive Exponential (AdEx) model (Gerstner and Brette, 2009), is another popular model that includes an exponential term to account for the adaptation of neuron firing rates over time. The Non-linear Integrate-and-Fire (*NLIF*) model (Jolivet et al., 2004), is a more complex model that includes non-linearity in the integration process. Other SNN models include the Integrate-and-Fire with Spike Frequency Adaptation (*IF* − *SFA*) model (Gigante et al., 2007), which incorporates a feedback mechanism that adjusts the neuron's firing rate based on its recent activity, the Theta Neuron model (McKennoch et al., 2009), which models the theta rhythm observed in the brain, the Hodgkin-Huxley (*HH*) model (Häusser, 2000), which is a biophysical model that includes multiple ion channels to capture the complex behavior of neurons, and the Quadratic-Integrate-and-Fire (*QIF*) model (Brunel and Latham, 2003), which is a more general model that allows for different types of threshold functions. Another widely used model in SNNs is the Izhikevich model (Izhikevich, 2004, 2007), which offers a balance between computational efficiency and biological plausibility. This model introduces a two-variable system that captures the dynamics of the neuron's membrane potential and recovery variable. The *IZH* model can replicate a wide range of spiking patterns observed in real neurons, making it suitable for various computational tasks. Additionally, the Spike Response Model (*SRM*; Gerstner, 2008), is another significant model in SNNs. The *SRM* focuses on capturing the post-spike response characteristics of neurons, which include the refractory period and the shape of the post-spike potential. By considering these response dynamics, the SRM provides a more detailed description of neuron behavior and enables the modeling of temporal effects in neural computations. Therefore, each of these models offers its own set of advantages and limitations, providing researchers and engineers with a diverse toolbox to simulate and understand the behavior of spiking neurons.

In addition to the challenges of selecting the most suitable SNN model, another challenge associated with SNNs is the need for specialized simulators to simulate the spiking behavior of neurons. One popular simulator used for SNNs is the Neural Engineering Framework (NEF; Stewart, 2012; Sanaullah et al., 2023b), which is a mathematical framework for designing and implementing neural models that are based on the principles of neuroscience. Despite the potential benefits of SNNs, there are still several challenges associated with their implementation. One of the most significant challenges is choosing the most appropriate SNN model for a given task (Stimberg et al., 2019; Sanaullah et al., 2022a,b).

This is particularly important for classification tasks, where accuracy and performance loss are critical. Another challenge is the computational complexity of simulating SNNs, which can require significant computational resources (Ayan et al., 2020). Therefore, choosing the appropriate SNN model and addressing the computational complexity associated with their simulation remain significant challenges that need to be addressed to realize their full potential.

In order to address this issue, a study was conducted to compare the performance, behavior, and spikes generation methodology of different SNN models using the same number of inputs and neurons. The challenge of determining the most suitable model was addressed by comparing the performance of different models, and the results were analyzed to determine the most effective one. Additionally, the study also compared the biological plausibility of the different neuron models used in the SNNs. The biological plausibility of a neuron model is an important factor to consider since it determines how well the model simulates the behavior of actual biological neurons. The study also compared the number of spiking operations required by each model to simulate the behavior of the same set of neurons. The results showed that the LIF models required the least number of spiking operations, while the *HH* model required the most. These findings could aid in selecting the most appropriate SNN model based on the specific requirements of the task, such as accuracy, biological plausibility, and computational efficiency. Overall, this study sheds light on the challenges and potential benefits of SNNs and their models. It highlights the importance of comparing different models to determine the most suitable and provides valuable insights for researchers and practitioners working in this area.

## 2. Background

Artificial neural networks are computational models inspired by the structure and function of the brain, aimed at replicating and understanding human abilities (Bishop, 1995). Unlike traditional artificial neural networks, which use continuous activation functions to transmit information, SNNs use a “spike train” representation, where the output of each neuron is a series of discrete spikes (Ghosh-Dastidar and Adeli, 2009a,c). This allows SNNs to model the precise timing and sequencing of neural activity, making them well-suited for tasks that require the processing of temporal patterns and spatiotemporal information (Zhang and Li, 2019). Therefore, these networks have found widespread use in machine learning tasks such as function approximation and pattern recognition (Le, 2013; Krizhevsky et al., 2017).

As AGI research progresses, these works will continue to serve as critical reference points for developing more efficient, intelligent, and adaptive artificial intelligence systems with real-world applications in diverse domains (Graves et al., 2008; Sanaullah et al., 2023a). Therefore, some critical research works have significantly contributed to the development of AGI through brain-inspired paradigms, highlighting their key contributions and implications for the future of artificial intelligence.

- Robust Spike-Based Continual Meta-Learning Improved by Restricted Minimum Error Entropy Criterion by Yang et al. (2022c): This pioneering study introduces a novel spike-based framework that uses entropy theory for online meta-learning in a recurrent SNN. MeMEE improves spike-based meta-learning performance, shown through tasks like autonomous navigation and working memory tests. It emphasizes the integration of advanced information theory in machine learning, offering new perspectives for spike-based neuromorphic systems.
- Heterogeneous Ensemble-based Spike-driven Few-shot Online Learning by Yang et al. (2022b): a novel spike-based framework employing entropy theory for few-shot learning in recurrent SNNs. The HESFOL model significantly enhances the accuracy and robustness of few-shot learning tasks in spiking patterns and the Omniglot dataset, as well as in few-shot motor control tasks. Our study emphasizes the application of modern entropy-based machine learning in state-of-the-art spike-driven learning algorithms.
- SAM: A Unified Self-Adaptive Multicompartmental Spiking Neuron Model for Learning with Working Memory introduced by Yang et al. (2022a): a self-adaptive spiking neuron model integrating spike-based learning with working memory. SAM shows efficient and robust performance across tasks like supervised learning, pattern classification, and meta-learning, making it valuable for neuromorphic computing in robotics and edge applications. It also offers insights into the biological mechanisms of working memory.
- Neuromorphic Context-dependent Learning Framework with Fault-tolerant Spike Routing (Yang et al., 2021a): This study introduces a scalable neuromorphic fault-tolerant hardware framework for event-based SNNs. It successfully learns context-dependent associations despite possible hardware faults, enhancing network throughput by 0.9–16.1%. The proposed system enables real-time learning, decision-making, and exploration of neuronal mechanisms in neuromorphic networks.

Furthermore, SNNs use mathematical models to simulate the behavior of biological neurons. These models capture the complex behavior of neurons, including the propagation and integration of electrical signals, as well as the refractory period during which a neuron cannot fire a new spike (Tavanaei et al., 2019; Wang et al., 2020). At a high level, SNNs are models of biological neural networks that simulate the behavior of neurons in the brain using mathematical equations. These equations capture the electrical and chemical activity that occurs within neurons and between them. The basic building block of an SNN is the spiking neuron, which models the behavior of a biological neuron that fires an action potential, or spike when it receives enough input. SNNs also use mathematical models to describe the connectivity between neurons. For example, a common model is the synapse model, which describes the strength and dynamics of the connections between neurons based on neurotransmitter release and reuptake (Deco et al., 2008). Overall, the mathematical models used in SNNs allow us to simulate the complex behavior of biological neural networks and understand how they process information. These models can also be used to develop new algorithms for machine learning and artificial intelligence, which are inspired by the way the brain processes information.

## 3. Results and discussion

Neuromorphic computing is a novel computing paradigm known for its low power consumption and high-speed response. Several notable research works in the field are worth mentioning. For instance, the “Smart Traffic Navigation System for Fault-Tolerant Edge Computing of Internet of Vehicles in Intelligent Transportation Gateway (Yang et al., 2023)” focuses on developing a fault-tolerant edge computing system for the Internet of Vehicle applications in intelligent transportation. “CerebelluMorphic” is a large-scale neuromorphic model and architecture designed for supervised motor learning (Yang et al., 2021c). Lastly, “BiCoSS” aims to create a large-scale cognitive brain with a multi-granular neuromorphic architecture (Yang et al., 2021b). These innovative works contribute to the advancement of neuromorphic computing and demonstrate its potential in various applications, from fault-tolerant edge computing to large-scale motor learning and cognition systems.

SNN models are typically built using mathematical equations that describe the behavior of spiking neurons. These equations take into account various factors such as the input current, membrane potential, and membrane time constant, to simulate the behavior of biological neurons. Each SNN model has its own set of equations that determine its behavior. In the study mentioned, different SNN models have been investigated. These include namely *LIF*, *NLIF*, *AdEx*, *IF* − *SFA*, *ThNeuron*, *HH*, *QIF*, *IZH*, and *SRM* models. Each of these models is implemented using the update method, which takes an input current and a time step and returns whether a spike has occurred or not. The update method uses the equations that describe the behavior of the spiking neurons to calculate the membrane potential of each neuron at each time step. If the membrane potential exceeds a certain threshold, a spike is generated and propagated to the next neurons. By investigating different SNN models, researchers can gain a better understanding of how the brain processes information and how to design more efficient and accurate artificial neural networks. SNN models have potential applications in various fields, including robotics, computer vision, and natural language processing.

Therefore, this study provides a comprehensive analysis of SNNs and their mathematical models and contributes to progress in the research discipline by addressing the challenges in implementing SNNs for classification tasks that demand high accuracy and low-performance loss using a simulational environment. The study compares the performance, behavior, and spike generation of different SNN models using the same inputs and neurons, providing valuable insights into the benefits and challenges of SNNs and their models. The study also quantifies the number of spiking operations required by each model to process the same inputs and output the same results, providing a comparative analysis of the computational efficiency of the models.

### 3.1. Types of SNN models

We initialize the instance variables for each model and generate random weights for each neuron. One of the simplest SNN models is the *LIF* model, which is described by Equation 1:


(1) $$\begin{array}{l} \tau_{m}\frac{dV}{dt}=-V\left(t\right)+I\left(t\right) \end{array}$$

where τ_*m*_ is the time constant, *V*(*t*) is the membrane potential of the neuron at time *t*, and *I*(*t*) is the input current at time *t*. The membrane potential is updated based on the Equation 2:


(2) $$\begin{array}{l} V\left(t\right)\leftarrow V\left(t\right)+\frac{-V\left(t\right)+I\left(t\right)}{\tau_{m}}\cdot dt \end{array}$$

If membrane potential reaches the threshold potential *V*_th_, the neuron fires a spike and the membrane potential is reset to the resting potential *V*_reset_, which is described in Equation 3:


(3) $$\begin{array}{l} \text{if }V\left(t\right)\geq V_{\text{th}},\text{then }V\left(t\right)\leftarrow V_{\text{reset}} \end{array}$$

A variation of the *LIF* model is the Non-Linear Integrate-and-Fire (*NLIF*) model, which takes into account the non-linear relationship between the membrane potential and the input current. The *NLIF* model is described by Equation 4:


(4) $$\begin{array}{l} \frac{dV}{dt}=\frac{-V+I}{\tau } \end{array}$$

where *V* is the membrane potential, *I* is the input current, τ is the membrane time constant, and $\frac{dV}{dt}$ represents the change in the membrane potential over time. The membrane potential is updated based on the Equation 5:


(5) $$\begin{array}{l} V\left(t\right)\leftarrow \{\begin{array}{l} V_{\text{reset}}+\alpha \cdot \left(V\left(t\right)-V_{\text{th}}\right)\text{ \& if }V\left(t\right)\geq V_{\text{th}}\\ V\left(t\right)\cdot \beta \text{ \& otherwise} \end{array} \end{array}$$

where *V*_reset_ is the resting potential, α and β are scaling factors, and *V*_th_ is the threshold potential. The membrane potential is multiplied by β if it is below the threshold, which models the leakage of current from the neuron over time. If the membrane potential reaches the threshold potential, the neuron fires a spike and the membrane potential is reset to the resting potential plus a scaled depolarization of the membrane potential (as described by Equation 6):


(6) $$\begin{array}{l} \text{if }V\left(t\right)\geq V_{\text{th}},\text{then }V\left(t\right)\leftarrow V_{\text{reset}}+\alpha \cdot \left(V\left(t\right)-V_{\text{th}}\right) \end{array}$$

Another SNN model is the *AdEX* model, which captures the dynamic behavior of spiking neurons more accurately than the *LIF* or *NLIF* models. The *AdEx* model is described by Equation 7:


(7) $$\begin{array}{l} \frac{dV}{dt}=\frac{-V+\tau_{m}I-V_{rheo}+{\text{Δ}}_{T}\exp\left(\frac{V-V_{spike}}{{\text{Δ}}_{T}}\right)}{\tau_{m}} \end{array}$$

where *V* is the membrane potential of the neuron, *I* is the input current, τ_*m*_ is the membrane time constant, *V*_*rheo*_ is the rheobase potential, Δ_*T*_ is the slope factor, and *V*_*spike*_ is the threshold potential at which the neuron fires an action potential. If *V* exceeds *V*_*spike*_, a spike is generated and *V* is reset to *V*_*reset*_.

The *IF* − *SFA* model is another SNN model that incorporates the adaptation of the firing rate of neurons in response to input stimuli. The model is based on the *LIF* model with an additional adaptation current, which modifies the membrane potential and firing rate of the neuron over time. The *IF* − *SFA* model is described by Equation 8:


(8) $$\begin{array}{l} \tau_{m}\frac{dV}{dt}=-V\left(t\right)+I\left(t\right)+w\left(t\right) \end{array}$$

where τ_*m*_ is the time constant, *V*(*t*) is the membrane potential of the neuron at time *t*, *I*(*t*) is the input current at time *t*, and *w*(*t*) is the adaptation current. The membrane potential is updated based on the following equations:


(9) $$\begin{array}{l} w\left(t\right)=w\left(t-1\right)+\frac{1}{\tau_{w}}\left(A\left(V\left(t-1\right)-V_{rest}\right)-w\left(t-1\right)\right)\text{Δ}t \end{array}$$

(10) $$\begin{array}{l} V\left(t\right)\leftarrow V\left(t-1\right)+\frac{-V\left(t-1\right)+I\left(t\right)-w\left(t\right)}{\tau_{m}}\cdot \text{Δ}t \end{array}$$

where Δ*t* is the time step, τ_*w*_ is the time constant for the adaptation current, *A* is the adaptation strength, and *V*_*rest*_ is the resting potential of the neuron. The adaptation current *w*(*t*) is computed based on the difference between the membrane potential and the resting potential, and is added to the input current in Equation 8. The membrane potential is updated based on Equation 10, where the adaptation current is subtracted from the input current.

If the membrane potential reaches the threshold potential *V*_*th*_, the neuron fires a spike and the membrane potential is reset to the resting potential *V*_*rest*_. The adaptation current is also updated according to Equation 11:


(11) $$\begin{array}{l} w\left(t\right)\leftarrow w\left(t\right)+b \end{array}$$

where *b* is a constant that represents the increase in the adaptation current after a spike is generated. The increase in the adaptation current leads to a decrease in the firing rate of the neuron over time, allowing the neuron to adapt to the input stimulus.

The *ThetaNeuron* model is a more complex model that incorporates the influence of a sinusoidal waveform, in addition to the input current and membrane potential. The *ThetaNeuron* model is described by Equation 12:


(12) $$\begin{array}{l} \frac{dV}{dt}=\frac{-V+I_{\text{syn}}+I_{\text{ext}}+I_{\text{theta}}}{\tau_{m}} \end{array}$$

where *V* is the membrane potential, *I*_syn_ is the synaptic input current, *I*_ext_ is the external input current, *I*_theta_ is the sinusoidal input current, and τ_*m*_ is the membrane time constant. The sinusoidal input current is described by Equation 13:


(13) $$\begin{array}{l} I_{\text{theta}}=I_{\text{thet}{\text{a}}_{\text{max}}}\cdot \sin\left(2\pi f_{\text{theta}}t+\phi \right) \end{array}$$

where *I*_thet_a__max__ is the amplitude of the sinusoidal input current, *f*_theta_ is the frequency of the theta rhythm, *t* is the time, and ϕ is the phase of the theta rhythm. The membrane potential is updated based on the Equation 14:


(14) $$\begin{array}{l} V\left(t\right)\leftarrow \{\begin{array}{l} V_{\text{reset}}\text{ \& if }V\left(t\right)\geq V_{\text{th}}\;V\left(t\right)\text{ \& otherwise} \end{array} \end{array}$$

where *V*_reset_ is the resting potential and *V*_th_ is the threshold potential. If the membrane potential reaches the threshold potential, the neuron fires a spike and the membrane potential is reset to the resting potential.

Furthermore, the *ThetaNeural* model incorporates a sinusoidal input current in addition to the synaptic and external input currents to update the membrane potential based on Equation 12. If the membrane potential reaches the threshold potential, the neuron fires a spike and the membrane potential is reset to the resting potential as described by the Equation 14. The *HH* and *QIF* models are fundamentally different from the *LIF*, *NLIF*, and *AdEx* models because they do not use differential equations to model the behavior of neurons. The *HH* model is a biophysical model that simulates the behavior of ion channels and currents in the neuron membrane. It is described by a system of differential equations that represent the time-dependent behavior of ion channels. The *QIF* model, on the other hand, is a simplified model that assumes that the neuron fires an action potential whenever its membrane potential crosses a threshold. The *QIF* model is described by Equation 15:


(15) $$\begin{array}{l} V_{i+1}=V_{i}+\frac{\text{Δ}t}{C}\left(g_{\text{syn}}\left(V_{\text{syn}}-V_{i}\right)+I_{\text{ext}}+I_{\text{noise}}\right) \end{array}$$

where *V*_*i*_ is the membrane potential of the neuron at time step *i*, *C* is the capacitance of the neuron, *g*_syn_ is the conductance of synaptic input, *V*_syn_ is the reversal potential of the synaptic input, *I*_ext_ is the external current input, and *I*_noise_ is the random noise input.

Additionally, the *Izhikevich* (*IZH*) model captures the dynamics of real neurons while being computationally efficient. It is described by a set of ordinary differential equations that govern the behavior of the neuron. The model consists of two main variables: the membrane potential, denoted by *v*(*t*), and a recovery variable, denoted by *u*(*t*). The *IZH* model is defined by the following equations,


(16) $$\begin{array}{l} \frac{dv}{dt}=0.04v^{2}+5v+140-u+I \end{array}$$

(17) $$\begin{array}{l} \frac{du}{dt}=a\left(bv-u\right) \end{array}$$

where *a*, *b*, and *I* are parameters that determine the behavior of the neuron. The parameter *I* represents the input current to the neuron, which can be thought of as the summation of all the incoming currents from other neurons or external sources. The terms 0.04*v*^2^ + 5*v* + 140 − *u* and *a*(*bv* − *u*) describe the dynamics of the membrane potential and the recovery variable, respectively. So, the update process of the implemented *IZH* model involves integrating these equations over time using numerical integration methods, such as Euler's method:


(18) $$\begin{array}{l} v\left(t+\text{Δ}t\right)=v\left(t\right)+\left(0.04v^{2}+5v+140-u+I\right)\cdot \text{Δ}t \end{array}$$

(19) $$\begin{array}{l} u\left(t+\text{Δ}t\right)=u\left(t\right)+\left(a\left(bv-u\right)\right)\cdot \text{Δ}t \end{array}$$

If the membrane potential *v*(*t*) exceeds a certain threshold, typically set to 30.0, the neuron is considered to have fired a spike. In that case, the membrane potential is reset to a specific value *c*, and the recovery variable is incremented by a fixed value *d*. This reset and increment simulate the after-spike behavior of the neuron. Hence, the update equations after a spike are:


(20) $$\begin{array}{l} v\left(t\right)=c \end{array}$$

(21) $$\begin{array}{l} u\left(t\right)=u\left(t\right)+d \end{array}$$

Therefore, the *IZH* model provides a computationally efficient yet biologically inspired representation of spiking neuron dynamics. By adjusting the parameters *a*, *b*, *c*, and *d*, different spiking patterns can be replicated, allowing for the modeling of a wide range of neuron behaviors.

In the *SRM*, each neuron has two variables: the membrane potential, denoted as *V*_*init*_, and the synaptic variables, denoted as *s*(*t*) and *r*(*t*). The membrane potential represents the electrical potential across the neuron's membrane, while the synaptic variables capture the post-synaptic response to incoming spikes. It is described by the equations:


(22) $$\begin{array}{l} \frac{ds}{dt}=-\frac{s}{\tau_{s}}+r \end{array}$$

(23) $$\begin{array}{l} \frac{dr}{dt}=-\frac{r}{\tau_{r}} \end{array}$$

(24) $$\begin{array}{l} \frac{dV}{dt}=-\frac{V-\text{input current}-\sum_{i}w_{i}s_{i}}{\tau_{s}} \end{array}$$

where τ_*s*_ and τ_*r*_ are the time constants for the synaptic and refractory dynamics, respectively. *s* and *r* are the synaptic variables, which decay exponentially over time. The input current represents the external input to the neuron, and $\underset{i}{\sum }w_{i}s_{i}$ represents the weighted sum of the incoming spikes from other neurons, where *w*_*i*_ is the weight associated with each connection and *s*_*i*_ is the corresponding synaptic variable of the pre-synaptic neuron. Therefore, the update process of the implemented *SRM* model involves integrating these equations over time using numerical integration methods:


(25) $$\begin{array}{l} s\left(t+\text{Δ}t\right)=s\left(t\right)+\left(-\frac{s\left(t\right)}{\tau_{s}}+r\left(t\right)\right)\cdot \text{Δ}t \end{array}$$

(26) $$\begin{array}{l} r\left(t+\text{Δ}t\right)=r\left(t\right)+\left(-\frac{r\left(t\right)}{\tau_{r}}\right)\cdot \text{Δ}t \end{array}$$

(27) $$\begin{array}{l} V\left(t+\text{Δ}t\right)=V\left(t\right)+\left(-\frac{V\left(t\right)-\text{input current}+\sum_{i}w_{i}s_{i}}{\tau_{s}}\right)\cdot \text{Δ}t \end{array}$$

Additionally, the *SRM* model includes a threshold potential *V*_th_ and a reset potential *V*_reset_. If the membrane potential reaches or exceeds the threshold *V*_th_, the neuron fires a spike, and the membrane potential is reset to *V*_reset_. The synaptic variables *s* and *r* are also incremented by 1.0 to represent the post-synaptic response to the spike.

However, it is possible to combine different models in a single network, as long as the models are compatible with each other and can be integrated seamlessly. For example, the *HH* model can be used to model the behavior of individual neurons in a network, while the *QIF* model can be used to model the behavior of the network as a whole. However, it is important to note that the *HH* and *QIF* models are much more complex and computationally intensive than the simpler *SNN* models and may not be suitable for all applications (Ma and Wu, 2007).

### 3.2. Dataset

Datasets play a crucial role in the development and evaluation of machine learning models, including spiking neural networks. They provide the necessary input for the models to learn and make predictions, and the quality and suitability of the dataset can significantly impact the model's performance. There are various types of datasets available for machine learning, including benchmark datasets, real-world datasets, and synthetic datasets. Each type of dataset has its advantages and disadvantages, and the choice of dataset depends on the task at hand and the goals of the study. The MNIST dataset and other benchmark datasets are commonly used for evaluating machine learning models, including spiking neural networks. However, these datasets are limited in terms of their complexity and do not necessarily reflect the challenges of real-world problems. In contrast, synthetic datasets can be tailored to specific tasks and can provide a more controlled environment for comparing different models. The synthetic dataset used in this study was designed to have two classes that are easily separable by a linear classifier, which allows for a straightforward evaluation of the performance of different models. Additionally, the synthetic dataset is more transparent in terms of the underlying data generation process, which can help in identifying the strengths and weaknesses of different models. For example, the MNIST dataset is a well-known benchmark dataset for image classification and the input images are preprocessed and normalized, the performance of each model is expected to be similar, due to the same parameters (tau, v_reset, v_th, alpha, n_neurons, and dt), and the same number of neurons (n_neurons) has been used for almost every model. Therefore, using a synthetic dataset can be a useful tool for comparing and evaluating different spiking neural network models, especially for tasks where real-world datasets are not readily available or do not provide enough complexity.

A synthetic dataset used for this study was generated using the following approach; Let *n*_*samples*_ = 1, 000, $x_{1}\sim N\left(0,1\right)$, $x_{2}\sim N\left(3,1\right)$, $X=\left[\begin{array}{cc} x_{1} & x_{2} \end{array}\right]$, $y=\left[\begin{array}{cc} 0_{n_{samples}} & 1_{n_{samples}} \end{array}\right]$, where 0_*n*_*samples*__ and 1_*n*_*samples*__ are the vectors of length *n*_*samples*_ filled with zeros and ones, respectively. To shuffle the dataset, let $indices=\left[\begin{array}{cccc} 0 & 1 & \cdots & 2n_{samples}-1 \end{array}\right]$, and apply a random permutation to *indices*. Then, let *X* and *y* be the arrays obtained by indexing *X* and *y* with the shuffled *indices*.

For example, the dataset contains a total of *n*_*samples*_ = 1, 000 samples, and two features, *x*_1_ and *x*_2_, were generated for each sample using normal distributions. Specifically, *x*_1_ was sampled from a normal distribution with a mean of 0 and a standard deviation of 1, denoted as N(0,1). On the other hand, *x*_2_ was sampled from a normal distribution with a mean of 3 and a standard deviation of 1, denoted as N(3,1). The feature matrix for all samples is denoted as *X* and is represented as follows:


(28) $$\begin{array}{l} X=\left[\begin{array}{cc} x_{1}^{\left(1\right)} & x_{2}^{\left(1\right)}\\ x_{1}^{\left(2\right)} & x_{2}^{\left(2\right)}\\ \vdots & \vdots \\ x_{1}^{\left(n_{\text{samples}}\right)} & x_{2}^{\left(n_{\text{samples}}\right)} \end{array}\right] \end{array}$$

The corresponding labels for the samples were created to form a binary classification problem. The label vector *y* has a length of 2*n*_*samples*_, containing *n*_*samples*_ zeros followed by *n*_*samples*_ ones. In mathematical notation:


(29) $$\begin{array}{l} y=\left[\begin{array}{cc} 0_{n_{samples}} & 1_{n_{samples}} \end{array}\right] \end{array}$$

Here, 0_*n*_*samples*__ represents a vector of length *n*_*samples*_ filled with zeros, and 1_*n*_*samples*__ represents a vector of length *n*_*samples*_ filled with ones. To randomize the dataset, a set of indices, denoted as *indices*, is created as follows:


(30) $$\begin{array}{l} indices=\left[\begin{array}{cccc} 0 & 1 & \cdots & 2n_{samples}-1 \end{array}\right] \end{array}$$

This array contains consecutive integers from 0 to 2*n*_*samples*_−1. Next, a random permutation is applied to the *indices* array to shuffle the dataset randomly. Finally, the feature matrix *X* and the label vector *y* are updated based on the shuffled indices. The elements of *X* and *y* are rearranged according to the new order provided by the shuffled *indices*. As a result of this process, the synthetic dataset with 1,000 samples, each having two features (*x*_1_ and *x*_2_) and corresponding binary labels. This dataset can be used to train and evaluate machine learning models for binary classification tasks.

### 3.3. Comparison

In this study, the performance of the different SNN neural models was explored using classification accuracy and performance loss as the performance metrics, where classification accuracy measures the percentage of correctly classified samples or data points by a model. Therefore, we used classification accuracy to evaluate how accurately each SNN model classified the input data based on the comparison of predicted spikes with the true labels. The accuracy is calculated as the mean of the element-wise equality comparison and then multiplied by 100 to obtain a percentage value. A higher accuracy indicates a better-performing model in terms of its ability to correctly classify the input patterns. Performance loss, on the other hand, quantifies the deviation or error of the model's predictions from the ground truth or desired output. It provides an indication of the model's ability to accurately represent the input data. In this study, the performance loss is calculated as the error rate, which demonstrates the percentage of misclassified samples. A lower error rate indicates a better-performing model with less deviation from the desired output. It is important to note that the specific definitions and measurements for classification accuracy and performance loss may vary depending on the context and objectives of the study. In our case, these metrics were chosen as they are commonly used in evaluating the performance of classification tasks and provide a straightforward assessment of the SNN models' capabilities.

It is important to note that, the proposed approach of this study does not implement a typical neural network with layers, connectivity, and learning mechanisms. Instead, it presents a collection of different single-neuron models and trains each model individually to classify a synthetic dataset. Each of the defined models (e.g., *LIF*, *NLIF*, *AdEx*, *HH*, etc.) represents a single-neuron model. These models are not interconnected in a multi-layer network, and there is no learning mechanism such as backpropagation or gradient descent. Therefore, using a single-neuron model have several benefits that make them useful for certain applications and provide clear comparisons.

- **Simplicity**: Single-neuron models are relatively simple compared to complex multi-layer neural networks. They provide a clear and intuitive understanding of how individual neurons respond to input stimuli and how their dynamics influence their behavior.
- **Insight into individual neuron behavior**: Each model focuses on a single neuron type and highlights specific properties and behaviors unique to that neuron. This allows researchers to study and compare the characteristics of different neurons in isolation.
- **Interpretability**: Due to their simplicity, single-neuron models are more interpretable. It is easier to analyze and understand the role of individual model parameters on the neuron's behavior.
- **Benchmarking**: Single-neuron models can serve as benchmarks for evaluating more complex neural network models. They provide a baseline to compare the performance of more sophisticated models in certain tasks, especially when the task can be effectively handled by a single neuron.
- **Model selection**: When faced with various neuron models, single-neuron simulations can help select the most suitable model for specific applications. Comparing the responses of different models to various inputs can aid in choosing the one that best captures the desired neuron behavior.
- **Biological plausibility**: Some of the single-neuron models, such as the Hodgkin-Huxley model, are biologically inspired and attempt to replicate the behavior of real biological neurons. These models help researchers explore and understand the mechanisms underlying neural dynamics.
- **Fast simulations**: Since single-neuron models have fewer parameters and computations compared to deep neural networks, simulations can be faster and computationally less demanding. This advantage is especially useful when exploring a wide range of parameters or conducting large-scale simulations.

**Figure 1:**
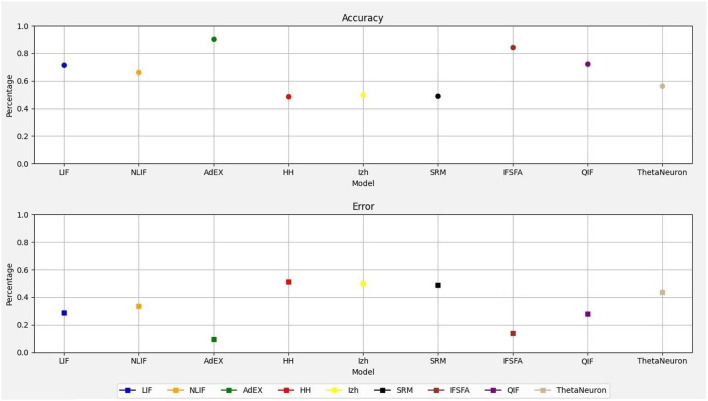
Comparison of accuracy and error rates for different neuron models.

Therefore, this study demonstrates how to simulate and evaluate the spike responses of different single-neuron models in response to a synthetic dataset. It does not implement a traditional neural network with layers and learning mechanisms. Figure 1 shows the accuracy and error rates of each neural model, allowing for a clear comparison of their performance. Among the models, the *AdEX* model demonstrated the highest accuracy, achieving an accuracy of 90.05% with an error rate of 0.10%. On the other hand, the *HH* model exhibited the lowest accuracy with error rates of 0.50 and 49.55% respectively, and the *SRM* model reveal the second lowest accuracy of 49.95% with an error rate of 0.50%. The *Izhikevich* model obtained an accuracy of 50.05% with an error rate of 0.50%. The simplest neural model, *LIF* achieved an accuracy of 71.20% with an error rate of 0.32% and the *NLIF* model achieved an accuracy of 66.55% with an error rate of 0.35%. However, the *IF* − *SFA* model also achieved an accuracy of 84.30% with an error rate of 0.14% and the *QIF* model achieved an accuracy of 70.70% with an error rate of 0.27%. Lastly, the *ThetaNeuron* model achieved an accuracy of 58.55% with an error rate of 0.42%. Overall, the AdEX model showed the highest accuracy, while the HH, IZH, and SRM models had the lowest accuracy. These accuracy and error rate values provide a quantitative assessment of the performance of each SNN model, allowing for a clear understanding of their relative capabilities in accurately classifying the input spiking patterns.

In addition to the accuracy and error rates, we wanted to understand how well each SNN model performed compared to the others. To do this, we used a metric called “performance loss.” Performance loss measures how much a particular SNN model's accuracy deviates from the accuracy of the best model on the dataset. In Table 1, you can see the performance loss of each model relative to the best model. Let's take the *LIF* model as the reference. The *LIF* model displayed a performance loss of –45.63% when compared to the *ThetaNeuron* model, indicating it performed 45.63% worse than the latter. In contrast, the *LIF* model had a performance loss of –9.36% relative to *NLIF*, meaning it performed 9.36% worse than *NLIF*. However, when comparing *LIF* to *AdEx*, it showed a performance loss of 20.08%, suggesting that it performed 20.08% better than *AdEx*.

**Table 1:** Performance loss of different SNN models.

**Model**	**Performance loss of each model relative to other models (%)**								
	**LIF**	**NLIF**	**AdEX**	**HH**	**Izh**	**SRM**	**IFSFA**	**QIF**	**Th.Nu**.
LIF	**X**	–45.63	–9.36	20.08	–40.27	–44.76	–44.90	–44.32	0.14
NLIF	–9.36	**X**	20.08	–40.27	–44.76	–44.90	–44.32	0.14	–33.17
AdEx	–25.12	–36.83	**X**	–75.51	–81.12	–81.30	–80.58	–24.95	–82.21
HH	28.71	22.04	43.02	**X**	–3.20	–3.30	–2.89	28.81	–3.82
Izhikevich	30.92	24.45	44.79	3.10	**X**	–0.10	0.30	31.01	–0.60
SRM	30.99	24.53	44.84	3.19	0.10	**X**	0.40	31.08	–0.50
IFSFA	30.71	24.23	44.62	2.81	–0.30	–0.40	**X**	30.81	–0.90
QIF	–0.14	–9.51	19.97	–40.46	–44.96	–45.10	–44.52	**X**	–45.83
*ThetaNeuron*	31.33	24.91	45.12	3.68	0.60	0.50	0.90	31.43	**X**

The table illustrates the classification accuracy of various single-neuron models, including *LIF*, *NLIF*, *AdEx*, *HH*, *IZH*, *SRM*, *IF* − *SFA*, *QIF*, and *ThetaNeuron*, on a synthetic dataset. The performance loss is calculated as the difference in accuracy between each model and the best-performing model among them. Lower performance loss values indicate better classification performance for a given model compared to others. This analysis provides insights into the diverse spiking behaviors and relative strengths of the different SNN models in the context of the classification task. X is used to indicate the model in a row that was compared with other models.

Similarly, the *HH* model exhibited a performance loss of –40.27% compared to *LIF*, indicating it performed 40.27% worse than *LIF*. The *Izhikevich* model had a performance loss of –44.76% relative to *LIF*, while the *SRM* model and the *IF* − *SFA* model had performance losses of –44.90 and –44.32% respectively, both compared to *LIF*. However, when comparing *LIF* to *QIF*, the performance loss was only 0.14%, suggesting that their performance was quite similar. Moving on to *NLIF*, it displayed a performance loss of –33.17% relative to *ThetaNeuron*, implying it performed 33.17% worse than *ThetaNeuron*. The performance loss of *NLIF* compared to *HH* and *Izhikevich* was –40.27 and –44.76% respectively, indicating its worse performance in both cases. Similarly, *NLIF* had a performance loss of –44.90 and –44.32% when compared to *SRM* and *IF* − *SFA* respectively. However, similar to *LIF*, *NLIF* showed a negligible performance loss of 0.14% when compared to *QIF*.

Lastly, let's consider *AdEx*, which served as the reference model. It displayed a substantial performance loss of –82.21% compared to *ThetaNeuron* and –75.51% compared to *HH*. Furthermore, its performance loss relative to *Izhikevich*, *SRM*, and *IF* − *SFA* was –81.12, –81.30, and –80.58% respectively. Surprisingly, *AdEx* showed a relatively lower performance loss of –24.95% when compared to *QIF*, indicating a better performance than *QIF*. This detailed analysis provides valuable realization into how each SNN model performed relative to the best model, helping us understand their strengths and weaknesses in the context of this dataset.

On the other hand, when we take the *LIF* model as the reference, we find that the *NLIF* model had a performance loss of –6.99%, which means it actually performed slightly better than *LIF*. The *HH* model had a performance loss of 29.28% relative to *LIF*, the *IF* − *SFA* model had a performance loss of 15.31%, and the *QIF* model had a performance gain of 0.70%, indicating it performed slightly better than *LIF*. The *ThetaNeuron* model had a performance loss of 20.01%.

These performance loss values demonstrate how much each SNN model deviates or misclassifies compared to the reference model mentioned. Negative values mean a smaller deviation, indicating better performance compared to the reference model. Positive values indicate a larger deviation, meaning worse performance compared to the reference model. Therefore, performance loss allows for a direct comparison of different SNN models relative to a selected reference model. Instead of focusing solely on absolute accuracy values, this metric provides insights into how well each model performs concerning a chosen benchmark, helping to identify the most suitable model for a particular task. Thus, measuring performance loss offers a valuable and efficient way to compare and evaluate the relative performance of different SNN models. It also complements traditional accuracy metrics and provides essential information for model selection, optimization, and understanding of the behavior of SNNs in practical applications.

#### 3.3.1. Network topology and training algorithm

To compare the intrinsic properties of different neural models and to ensure a fair comparison, as a result, we used a common network topology and synaptic weight configuration for all models. The network topology refers to the arrangement of neurons and their connections in the network. In our case, we used a consistent topology with 1,000 neurons. However, it's important to note that the choice of network topology can significantly impact the performance of SNN models. Different network structures, such as random (Polk and Boudreaux, 1973), small-world (Fell and Wagner, 2000), or scale-free (Goh et al., 2002) networks, can exhibit different dynamical behaviors and information processing capabilities. Optimizing the network topology based on the specific requirements of a given task or problem can enhance the performance of SNN models. Similarly, synaptic weights represent the strength of connections between neurons. In our study, we used random weights for each model. However, the optimization of synaptic weights is crucial for achieving desired network behavior. Adjusting the synaptic weights can modulate the influence of one neuron on another and control the overall dynamics of the network. Techniques such as Hebbian learning (Kosko, 1986), spike-timing-dependent plasticity (STDP; Dan and Poo, 2004), or other learning rules can be employed to optimize the synaptic weights based on specific learning objectives or data patterns. Therefore, in this study, we focused on comparing the intrinsic properties of different SNN models, the optimization of network topology and synaptic weights is an important aspect that could be explored in future studies. By fine-tuning these parameters, it is possible to further enhance the performance and capabilities of SNN models, making them more suitable for specific applications or tasks.

Furthermore, the primary focus of this research study was specifically focused on comparing the behavior and performance of different SNN neural models without involving specific training algorithms. This choice allowed us to evaluate the inherent characteristics of each model in a controlled setting. Therefore, by executing the SNN models with the same inputs and analyzing their spike generation patterns, we aimed to gain a deep understanding of the fundamental properties of each model, such as their spike response dynamics and computational capabilities. This approach enabled researchers and developers to compare how each model processed and encoded information in the form of spiking activity. Furthermore, the theoretical basis for these performance measurements lies in the goal of accurately representing and classifying input patterns within the context of SNNs. Classification accuracy and performance loss provide quantitative measures to assess how well and accurately a model captures and interprets the information contained in the input spiking patterns.

However, it's important to note that training algorithms play a crucial role in optimizing SNN models for specific tasks or learning objectives. Different training algorithms can be employed to adjust the synaptic weights and optimize the network's performance. But the choice of training algorithm can significantly impact the performance and capabilities of SNN models. Some models may perform better than others when subjected to a specific training algorithm, while their performance may differ when another algorithm is used. Thus, the selection of a suitable training algorithm depends on the specific task at hand and the desired learning objectives. Therefore, future research can explore the impact of different training algorithms on the performance of the compared SNN models. To provide a more comprehensive analysis by evaluating the models' performance under various training scenarios, researchers can gain a deeper understanding of the interplay between model architectures and learning algorithms, ultimately leading to more informed design choices for specific applications or tasks.

#### 3.3.2. Spiking activity

The spiking activity of each neural model is crucial because it reflects the dynamic behavior of neurons in SNNs. Unlike traditional artificial neural networks, which rely on continuous activation functions, SNNs operate based on the generation of discrete spikes. Understanding the spiking activity of different neural models provides insights into their temporal characteristics, spike patterns, firing rates, and the information processing capabilities of the networks. Therefore, in this study, we investigate different SNN models and these models are used to simulate spiking activity in neurons under different conditions. For example, the *LIF* and *NLIF* models use a simple leaky integrate-and-fire approach to generate spikes based on the input current, while the *AdEx* model includes an adaptation current that changes over time. The *HH* model is based on the Hodgkin-Huxley equations and includes voltage-gated ion channels that contribute to spike generation. Each of these models is characterized by a set of parameters that define the behavior of the neuron. For example, the membrane time constant, membrane resistance, and spike threshold are important parameters for the *LIF* and *NLIF* models. The *AdEx* model includes additional parameters for the adaptation current, such as the time constant and the subthreshold and spike-triggered conductances. The *HH* model includes parameters for the maximum conductances of different ion channels, their reversal potentials, and the gating variables that control their activation and inactivation.

**Figure 2:**
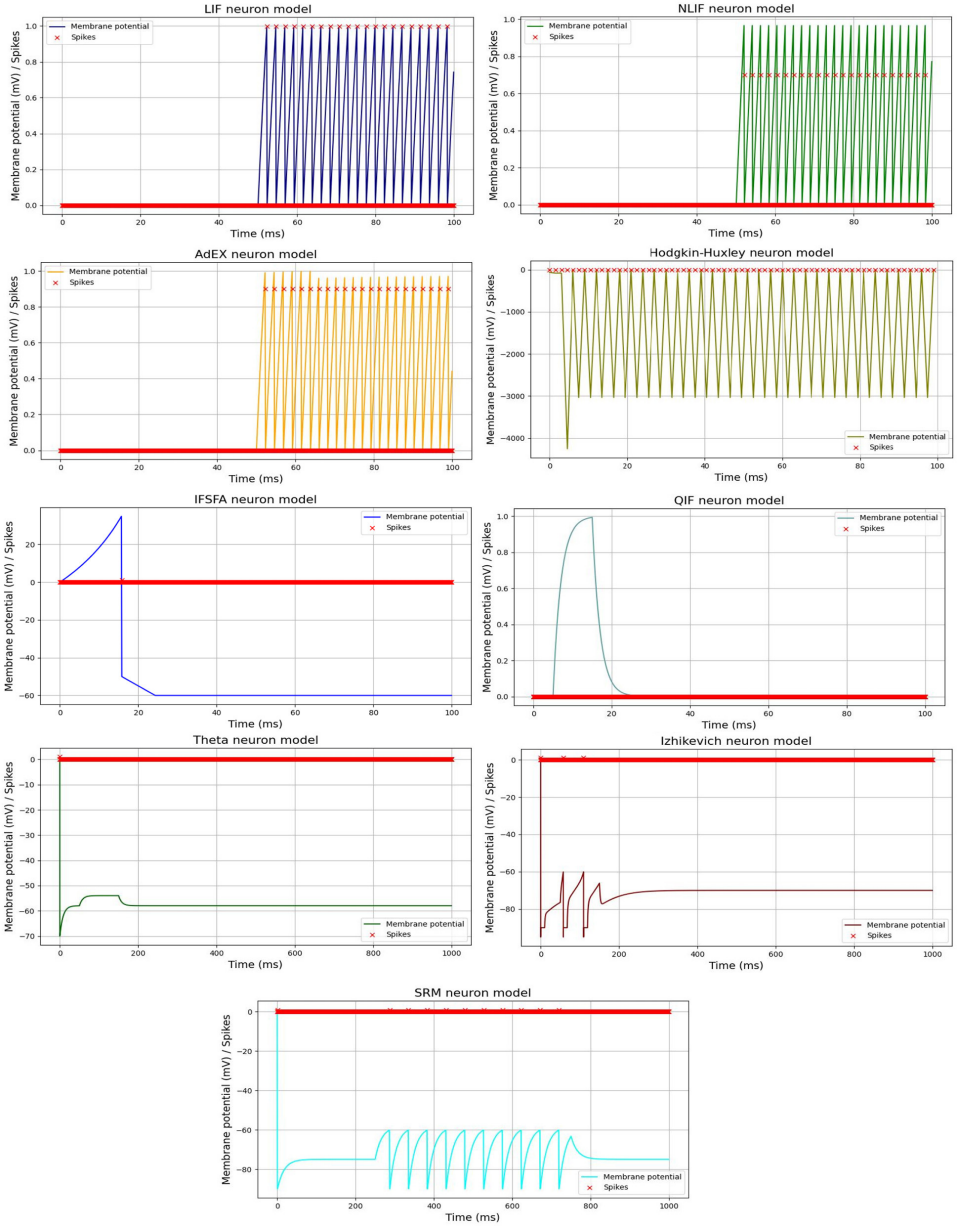
Spiking activity of different SNN models in response to an input current pulse. Each line or curve represents the membrane potential of a specific neuron model, while the red “x” markers indicate the occurrences of spikes. Each model exhibits unique dynamics, producing distinct patterns of spiking activity.

Furthermore, we visualized the spiking activity of the neurons in each SNN model over time duration of each time interval in a simulation or numerical computation. For example, Huxley neuron model, the time step determines how frequently the state variables of the system (e.g., membrane potential and gating variables) are updated based on the differential equations. Figure 2, shows the spiking activity of each neural model in response to an input current pulse, and Table 2, represents the parameter values used for each model spiking activity comparison. The input current pulse (depicted by the horizontal bar) is applied to all the models at the same time points. As the input current is integrated by each model, their respective membrane potentials rise. Once the threshold potential is reached, each neuron generates a spike, and the membrane potential is reset to its resting potential (*V*_*reset*_). A refractory period is applied to simulate the temporary inactivity of the neurons after spiking. Different models may exhibit various spiking patterns, response times, and numbers of spikes depending on their unique dynamics and parameters. The combined Figure 2 allows for a direct visual comparison of how each model responds to the same input current pulse.

**Table 2:** Parameter values employed for each model.

**Parameters**	**Parameter values used for each-model**								
	**LIF**	**NLIF**	**AdEX**	**HH**	**Izh**	**SRM**	**IFSFA**	**QIF**	**Th.Nu**.
Time step	0.1	0.1	0.1	1.5	0.1	0.5	0.1	0.1	0.1
Time constant	10	10	10	X	20	10	X	X	20
Membrane resistance	1.0	1.0	1.0	X	10	5.0	1.0	X	X
Threshold value	1.0	1.0	1.0	–66	–60	–60	35	1.0	–50
Reset membrane potential (MP)	0.0	0.0	0.0	0.0	–90	–90	–60	0.0	–70
Initial potential	0.5	0.5	0.5	–65	–90	–90	–90	0.5	–70
Refractory period	X	X	X	X	10	1.0	X	2	X
Leak conductance	0.1	0.1	0.1	0.3	X	0.01	X	X	X
Adaptation time constant	30	30	30	X	X	X	200	X	X
Adaptation conductance	0.001	0.001	0.001	X	X	X	0.01	X	X
Adaptation increment	0.01	0.01	0.01	X	X	X	–0.1	X	X
Membrane capacitance	X	X	X	1.0	X	5.0	100	0.1	X
Sodium conductance	X	X	X	120	X	X	X	X	X
Potassium conductance	X	X	X	36	X	X	X	X	X
Sodium reversal	X	X	X	50	X	X	X	X	X
Potassium reversal	X	X	X	–82	X	X	X	X	X
Leak reversal	X	X	X	–84.4	X	X	X	X	X
Recovery variable (RV)	X	X	X	X	0.02	X	X	X	X
Sensitivity (RV/MP)	X	X	X	X	0.2	X	0.7	X	X
After-spike reset	X	X	X	X	–95	X	–50	X	X
After-spike reset (RV/AV)	X	X	X	X	6.0	X	100	X	X
Input conductance	X	X	X	X	X	1.5	X	X	1.5
Synaptic time constant	X	X	X	X	X	5.0	X	X	X
Synaptic reversal potential	X	X	X	X	X	0.01	X	X	X
Synaptic weight	X	X	X	X	X	0.09	X	X	X

In general, the visualization of spiking activity over time is a commonly used technique for analyzing and comparing different SNN models. For example, the *LIF* model is a simplified version of the *NLIF* model, where there is no subthreshold adaptation.

***LIF*** **neural model:**

The differential equation used in generating spike activity for the membrane potential *V*_*lif*_ in the *LIF* model is given by Equation 31:


(31) $$\begin{array}{l} dV=\frac{-V_{lif}\left[i-1\right]+\left(R_{m}*I\left[i-1\right]\right)/g_{leak}}{\tau } \end{array}$$

Where *dV* is the change in membrane potential and *V*_*lif*_[*i*], is the membrane potential at the time step *i*. *R*_*m*_ is the membrane resistance. *I*[*i* − 1] is the input current at the time step *i* − 1. *g*_*leak*_ is the leak conductance and τ is the membrane time constant. So, if the membrane potential *V*_*lif*_[*i*] crosses the spike threshold *V*_*th*_, a spike is generated (*Spikes*_*lif*_[*i*] = 1), and the membrane potential is reset to *V*_*reset*_.

***NLIF*** **neural model:**

The *NLIF* model is an extension of the classical LIF model with subthreshold adaptation. It includes a nonlinearity in the update of the membrane potential. The differential equation used in generating spike activity for the membrane potential *V*_*nlif*_ in the NLIF model is given by Equation 32:


(32) $$\begin{array}{l} dV=\frac{-V_{nlif}\left[i-1\right]+\frac{R_{m}*I\left[i-1\right]}{g_{leak}}+V_{nlif{\left[i-1\right]}^{2}}}{\tau } \end{array}$$

Where the variables have the same meaning as in the LIF model. Accordingly, If the membrane potential *V*_*nlif*_[*i*] crosses the spike threshold *V*_*th*_, a spike is generated (*Spikes*_*nlif*_[*i*] = 0.7), and the membrane potential is reset to *V*_*reset*_.

***AdEx*** **neural model:**

The *AdEx* model is a more biologically detailed model that includes adaptive exponential conductances. It captures the spike-frequency adaptation phenomenon. The differential equations for the membrane potential *V*_*adex*_ and the adaptation variable *w*_*adex*_ in the *AdEx* model are given by these equations,


(33) $$\begin{array}{l} dV=\frac{-V_{adex}\left[i-1\right]+\frac{R_{m}*I\left[i-1\right]}{g_{leak}}+w_{adex}\left[i-1\right]*\text{exp}\left(\frac{V_{adex}\left[i-1\right]-V_{th}}{\tau }\right)}{\tau } \end{array}$$

(34) $$\begin{array}{l} V_{adex}\left[i\right]=V_{adex}\left[i-1\right]+dV*dt \end{array}$$

(35) $$\begin{array}{l} dw=\frac{a*\left(V_{adex}\left[i-1\right]-V_{reset}\right)-w_{adex}\left[i-1\right]}{\tau_{w}} \end{array}$$

(36) $$\begin{array}{l} w_{adex}\left[i\right]=w_{adex}\left[i-1\right]+dw*dt \end{array}$$

Here, *dV* is the change in membrane potential, *V*_*adex*_[*i*] is the membrane potential, and *w*_*adex*_ is the adaptation variable at time step *i*. *R*_*m*_ is the membrane resistance and τ_*w*_ is the adaptation time constant. Where *a* is the subthreshold adaptation conductance and *b* is the spike-triggered adaptation increment. If the membrane potential *V*_*adex*_[*i*] crosses the spike threshold *th*, a spike is generated (*Spikes*_*adex*_[*i*] = 0.9), and both the membrane potential and the adaptation variable are reset accordingly. These models demonstrate different levels of complexity in simulating the behavior of neurons. Because the NLIF model introduces a nonlinear update of the membrane potential, the LIF model simplifies it to linear, and the AdEx model adds spike-frequency adaptation to capture more biological realism.

***HH*** **neural model:**

The *HH* model is a biophysical model that describes the behavior of excitable cells, such as neurons. It is based on the dynamics of ion channels and membrane currents. The *HH* model includes four state variables, *V* (membrane potential), *n* (activation variable for potassium), *m* (activation variable for sodium), and *h*(inactivation variable for sodium). So, the differential equations used for the calculation of spiking activity for the state variables in the HH model by the following equations,


(37) $$\begin{array}{l} \frac{dn}{dt}=\alpha_{n}\left(V\right)*\left(1-n\right)-\beta_{n}\left(V\right)*n \end{array}$$

(38) $$\begin{array}{l} \frac{dm}{dt}=\alpha_{m}\left(V\right)*\left(1-m\right)-\beta_{m}\left(V\right)*m \end{array}$$

(39) $$\begin{array}{l} \frac{dh}{dt}=\alpha_{h}\left(V\right)*\left(1-h\right)-\beta_{h}\left(V\right)*h \end{array}$$

Where *V* is the membrane potential. *n*, *m*, and *h* are gating variables representing the probabilities of the corresponding ion channels being open. [α_*n*_(*V*), β_*n*_(*V*)], [α_*m*_(*V*), β_*m*_(*V*)], and [α_*h*_(*V*), β_*h*_(*V*)] are voltage-dependent rate functions that control the opening and closing of the ion channels. And the ionic currents in the *HH* model are described by:


(40) $$\begin{array}{l} I_{Na}=g_{Na}*m^{3}*h\left(V-E_{Na}\right) \end{array}$$

(41) $$\begin{array}{l} I_{K}=g_{K}*n^{4}\left(V-E_{K}\right) \end{array}$$

(42) $$\begin{array}{l} I_{L}=g_{L}\left(V-E_{L}\right) \end{array}$$

Here *I*_*Na*_, *I*_*K*_, and *I*_*L*_ represent sodium current, potassium current, and leak current respectively. where *g*_*Na*_, *g*_*K*_, and *g*_*L*_ are the maximum conductances of the corresponding ion channels and *E*_*Na*_, *E*_*K*_, and *E*_*L*_ are the reversal potentials for sodium, potassium, and the leak conductance, respectively.

Thus, the total membrane current *I*_*m*_ is the sum of the ionic currents and the input current *I* divided by the membrane capacitance *C*_*m*_:


(43) $$\begin{array}{l} I_{m}=\frac{I+I_{Na}+I_{K}+I_{L}}{C_{m}} \end{array}$$

If the membrane potential *V* crosses the spike threshold *th*, a spike is generated (*Spikes*_*hh*_[*i*] = 1, and the membrane potential is reset to *V*_*reset*_. The gating variables *n*, *m*, and *h* are also updated using the rate functions accordingly. Therefore, the HH model is more biophysically detailed than the previously explained neuron models, as it considers the dynamics of multiple ion channels and their interactions in generating action potentials.

***IFSFA*** **neural model:**

The *IFSFA* model is designed to capture the phenomenon of neurons firing at different frequencies depending on the input current and their previous spiking activity. The *IFSFA* model includes two state variables, *V*_*ifsfa*_ (membrane potential) and *U*_*ifsfa*_ (adaptation variable). Following equations are used to calculate the spiking activity of the *IFSFA* model.


(44) $$\begin{array}{l} \frac{dV_{ifsfa}}{dt}=\frac{R_{m}\left(0.04*V_{ifsfa}^{2}+140-U_{ifsfa}+1\right)}{C_{m}} \end{array}$$

(45) $$\begin{array}{l} \frac{dU_{ifsfa}}{dt}=a\left(K*\left(V_{ifsfa}-V_{r}\right)-U_{i}fsfa\right) \end{array}$$

Where *V*_*r*_ is the resting membrane potential, *K* is a sensitivity parameter of the membrane potential, and *a* is the subthreshold adaptation conductance. The rest of the variables have the same meaning as defined above. The adaptation variable *V*_*ifsfa*_ is used to implement spike-frequency adaptation. When the membrane potential *V*_*ifsfa*_ crosses the threshold *V*_*peak*_, a spike is generated (*Spikes*_*ifsfa*_[*i*] = 1), and the membrane potential is reset to a lower value *C* Additionally, the adaptation variable *U*_*ifsfa*_ is increased by the after-spike reset value *d*. So, If the membrane potential *V*_*ifsfa*_ falls below the resting membrane potential *V*_*r*_, it is reset to *V*_*r*_ to prevent it from becoming negative. Overall, the IFSFA model is a simple yet effective way to incorporate spike-frequency adaptation into the integrate-and-fire neuron model to better mimic certain aspects of real neuron behavior.

***QIF*** **neural model:**

The QIF model incorporates a quadratic term to capture the nonlinearity of the neuron's behavior near the spike threshold. That includes three main components: the membrane potential *V*_*qif*_, the spike threshold *V*_*th*_, and the reset potential *V*_*reset*_. Below described equation is used for calculating the spiking activity of the *QIF* model based on Table 2 shown values,


(46) $$\begin{array}{l} \frac{dV_{qif}}{dt}=\frac{-V_{qif}+\sqrt{C_{m}}*\sqrt{I}}{\tau_{ref}} \end{array}$$

The quadratic term, $\sqrt{C_{m}}*\sqrt{I}$, introduces nonlinearity to the model. The membrane potential *V*_*qif*_ approaches the spike threshold more rapidly as the input current *I* increases. When the membrane potential *V*_*qif*_ crosses the spike threshold *V*_*th*_, a spike is generated (*Spikes*_*qif*_[*i*] = 1), and the membrane potential is reset to *V*_*reset*_. Additionally, a refractory period is initiated to prevent immediate spiking after a spike. The refractory period is represented by the variable *ref*_*qif*_, which is initialized to zero and is set to $\frac{\tau_{r}ef}{dt}$ when a spike occurs. During the refractory period, the membrane potential remains at the reset potential. So, If the refractory period *ref*_*qif*_ is greater than zero, the membrane potential is held at the reset potential *V*_*reset*_ until the refractory period ends. Thus, it is a model that provides a simple yet effective way to introduce nonlinearity near the spike threshold, which allows it to capture certain behaviors of spiking neurons more accurately.

***ThetaNeuron*** **neural model:**

The *ThetaNeuron* model incorporates a threshold potential θ to generate spikes. It is designed to represent neurons that exhibit spiking activity when the membrane potential reaches a specific threshold. It includes two main components: the membrane potential *V*_θ_ and the threshold potential θ. Therefore, to calculate the spiking activity of the *ThetaNeuron* model based on Equation 47 and using the values shown in Table 2 for demonstration, the generated plot can be observed in Figure 2.


(47) $$\begin{array}{l} \frac{dV_{theta}}{dt}=\frac{-\left(V_{theta}-Vr\right)+g\left(\theta -V_{theta}\right)+I}{\tau } \end{array}$$

The *ThetaNeuron* model equation includes three terms that affect the change in membrane potential:

− (*V*_*theta*_ − *Vr*): This term represents the difference between the membrane potential *V*_*theta*_ and the reset potential *V*_*r*_. It drives the membrane potential back toward the reset potential after a spike.
2. *g*(θ − *V*_*theta*_: This term represents the input conductance *g* multiplied by the difference between the threshold potential θ and the current membrane potential *V*_*theta*_. When the membrane potential approaches the threshold potential, this term drives the potential toward the threshold, potentially leading to a spike.
3. *I*: This term represents the input current at the time step *i* − 1. It provides external input to the neuron and can influence the neuron's spiking behavior.

Therefore, the *ThetaNeuron* model is a useful model for simulating neurons that fire action potentials when the membrane potential reaches a specific threshold. It is commonly used in neural network simulations and can be adapted to capture various firing patterns by adjusting the model's parameters.

***Izhikevich*** **neural model:**

The *Izhikevich* model is a two-dimensional simplified neuron model proposed by Eugene M. Izhikevich. It captures the spiking behavior of neurons with a reduced set of variables and equations. The Izhikevich model includes two main components: the membrane potential *V*_*izh*_ and the recovery variable *u*. The following differential equations are used for the calculation of spiking activity for the membrane potential *V*_*izh*_ and the recovery variable *u* in the *Izhikevich* model are given by:


(48) $$\begin{array}{l} \frac{dV_{izh}}{dt}=\frac{R_{m}\left(0.04*V_{izh}^{2}+5*V_{izh}+140-u+I\right)}{\tau } \end{array}$$

(49) $$\begin{array}{l} \frac{du}{dt}=a\left(b*V_{izh}-u\right) \end{array}$$

Where *a* is the recovery variable time scale, *b* is the sensitivity of the recovery variable, and *I* is the input current at time step *i* − 1. The first Equation 48 represents the evolution of the membrane potential *V*_*izh*_ based on the input current *I* and the recovery variable *u*. The second Equation 49 represents the evolution of the recovery variable *u* based on the membrane potential *V*_*izh*_ and its own dynamics. Therefore, the variable *in*_*refractory*_*period* is used to implement a refractory period after a spike. If *in*_*refractory*_*period* is greater than zero, the neuron is in a refractory period, and the membrane potential remains at the reset potential until the refractory period ends. This refractory period is implemented to prevent immediate spiking after a spike. Overall, the Izhikevich neuron model is widely used in computational neuroscience and artificial neural networks due to its simplicity and versatility in capturing various neuron behaviors.

***SRM*** **neural model:**

The SRM model incorporates synaptic dynamics to simulate post-synaptic responses following spikes. It models the dynamics of the membrane potential *V*_*srm*_ and the post-synaptic response variable *s* to simulate the neuron's spiking behavior and synaptic interactions. We used the following equations for generating the spiking activity of the SRM model using parametric values shown in Table 2:


(50) $$\begin{array}{l} \frac{ds}{dt}=\frac{\left(-s+A*spikes_{srm}\right)}{\tau_{s}} \end{array}$$

(51) $$\begin{array}{l} \frac{dV_{srm}}{dt}=\frac{-\left(V_{srm}-V_{r}\right)+g\left(\theta -V_{srm}\right)+s*A\left(E_{s}-V_{srm}\right)+I}{\tau *C_{m}} \end{array}$$

Where τ_*s*_ is the synaptic time constant, *A* is the synaptic weight representing the strength of the synapse, θ is the threshold potential, *g*_*L*_ is the leak conductance, *E*_*s*_ is the synaptic reversal potential, and *V*_0_ is the initial potential (set to a value higher than θ for spiking). The rest of the variables have the same meaning as defined above. Therefore, the first Equation 50 represents the dynamics of the post-synaptic response variable *s*, which captures the synaptic conductance changes following spikes from other neurons. It decays toward zero with a time constant τ_*s*_ and is updated based on the occurrence of spikes from the neuron (*spikes*_*srm*_).

The second Equation 51 represents the dynamics of the membrane potential *V*_*srm*_ based on the input current *I*, the synaptic conductance *s*, and the leak conductance *g*_*L*_. The term (*s* * *A*(*E*_*s*_ − *V*_*srm*_) contributes to the synaptic current. When the membrane potential *V*_*srm*_ crosses the threshold θ, a spike is generated (*spikes*_*srm*_[*i*] = 1), and the membrane potential is reset to the reset potential *V*_*r*_. Additionally, to model the leak conductance, the equation −*V*_*srm*_ + *V*_0_ is integrated over time, and the leak conductance *g*_*L*_ is applied every τ_*ref*_ time steps. Because of that, the SRM neuron model is a valuable tool for computational neuroscience and neural network simulations that involve synaptic interactions.

Thus, by simulating the spiking activity of various neural network models, researchers can gain a clear understanding of the mechanisms underlying neural computation and communication. For instance, they can investigate how different input patterns affect the firing rate and temporal precision of spike trains or how different neuromodulators or pharmacological agents impact the behavior of ion channels and other membrane proteins. Such study and analysis help researchers and developers to understand how different models simulate the behavior of biological neurons. It allows for the exploration of various coding schemes, spike-timing-dependent plasticity, and other mechanisms that play a significant role in information processing in the brain. Additionally, studying the spiking activity can provide insights into the computational efficiency and resource requirements of different models, aiding in the selection of the most suitable model for specific applications. By examining the spiking activity of each neural model, researchers can gain a deeper understanding of how these models perform and behave, ultimately leading to advancements in SNNs and their applications.

#### 3.3.3. Computational complexity

Each neuron model is a mathematical description of how neurons work, and each model has its own set of equations that are used to simulate the behavior of neurons. These equations involve mathematical operations such as addition, multiplication, and exponentiation, which are computationally expensive. Therefore, analyzing the computational complexity of each model is updating the neuron state at every time step, but their implementations are different. For example, the *LIF* model uses a simple thresholding rule, while the *NLIF* model uses a non-linear function to update the neuron state. Thus, analyzing the computational complexity of each model, e.g., by counting the number of operations required to update the neuron state in one time step, and comparing them.

In our study, the num_ops attribute is used to track the number of operations required to initialize and update each model. Initialization refers to the process of setting up the model with the appropriate parameters and data structures while updating refers to the process of updating the model based on new input data. By adding up the number of operations required for initialization and updates, the proposed algorithm is able to estimate the total computational complexity of each model. This can be useful for comparing the relative efficiency of different models and for identifying bottlenecks in the training process.

Based on our study results, here's a detailed analysis of the computational complexity of each neuron model:

1. ***LIF*** **model**
  - Mathematical description: The *LIF* model updates the neuron's membrane potential (*v*) over time based on the input current (*I*) and a time constant (τ).
  - Update rule: $\frac{dv}{dt}$ = $\frac{\left(-v+I\right)}{tau}$.
  - Computational complexity: The *LIF* model involves one multiplication, one subtraction, and one comparison in each update. Therefore, it requires 3, 003 operations to update the neuron state in a one-time step.
2. ***NLIF*** **model**
  - Mathematical description: The *NLIF* model extends the *LIF* model by introducing a non-linear adaptation term (w). The membrane potential (*v*) and adaptation (*w*) variables are updated based on the input current (*I*) and different time constants (τ, τ_*w*_).
  - Update rule: $\frac{dv}{dt}$ = $\frac{\left(-v+I+\left(weights.T,w\right)\right)}{\tau }$ and $\frac{dw}{dt}$ = $\frac{\left(a*\left(v-v_{reset}\right)-w\right)}{\tau_{w}}$.
  - The *NLIF* model involves one multiplication, one subtraction, one comparison, two additions, and two multiplications for the non-linear in each update. It requires 5, 092 operations to update the neuron state in one-time step.
3. ***AdEx*** **model**
  - Mathematical description: The *AdEx* model includes adaptive properties and exponential adaptation. It updates the neuron's membrane potential (*v*) and includes an adaptation current (*w*) that exhibits exponential dynamics based on the input current (*I*) and various model parameters (τ_*m*_, *v*_*rheo*_, *v*_*spike*_, and *delta*_*T*_).
  - Update rule: $\frac{dv}{dt}$ = $\frac{(-v+\tau_{m}*I-v_{rheo}+\delta_{T}*exp\left(\frac{\left(v-v_{spike}\right)}{\delta_{T}}\right)}{\tau_{m}}$.
  - Computational complexity: The *AdEx* model involves one multiplication, and four additions for the exponential dynamics in each update. It requires 4, 305 operations to update the neuron state in one-time step.
4. ***HH*** **model**
  - Mathematical description: The *HH* model is a biophysically detailed model that describes the behavior of voltage-gated ion channels in neurons. It updates the membrane potential (*v*) and gating variables (*n*, *m*, and *h*) based on the input current (*I*) and various model parameters (α_*n*_, β_*n*_, α_*m*_, β_*m*_, α_*h*_, and β_*h*_).
  - Update rule: The HH model involves four differential equations for $\frac{dv}{dt}$, $\frac{dn}{dt}$, $\frac{dm}{dt}$, and $\frac{dh}{dt}$.
  - Computational complexity: The *HH* model involves 18 additions, 15 multiplications, and three divisions in each update. However, it requires only 45 operations to update the neuron state in one-time step, which makes the *HH* model, the least computationally expensive among the considered models due to its simple update equations.
5. ***IFSFA*** **model**
  - Mathematical description: The *IF* − *SFA* model includes a spike-frequency adaptation mechanism and updates the membrane potential (*v*) and adaptation variable (*w*) based on the input current (*I*) and various model parameters (τ_*m*_, τ_*w*_, *a*, *b*, and δ_*T*_).
  - Update rule: $\frac{dv}{dt}$ = $\frac{\left(-v+\delta_{T}*exp\left(\frac{\left(v-v_{th}\right)}{\delta_{T}}\right)+\left(weights.T,w\right)+I\right)}{\tau_{m}}$ and $\frac{dw}{dt}$ = $\frac{\left(a*\left(v-v_{reset}\right)-w\right)}{\tau_{w}}$.
  - Computational complexity: The *IF* − *SFA* model involves two multiplications, two additions, one subtraction, and one comparison in each update. It requires 2, 260 operations to update the neuron state in one-time step.
6. ***QIF*** **model**
  - Mathematical description: The *QIF* model is a quadratic non-linearity and updates the membrane potential (*v*) based on the input current (*I*) and model parameters (τ and β).
  - Update rule: $\frac{dv}{dt}$ = $\frac{\left(-v+\beta *v^{2}+I\right)}{\tau }$.
  - Computational complexity: The *QIF* model involves one multiplication, one addition, one comparison, and a reset operation in each update. It requires 1, 018 operations to update the neuron state in one-time step.
7. ***ThetaNeuron*** **model**
  - Mathematical description: The *ThetaNeuron* model introduces phase dynamics and oscillatory behavior to neurons. It updates the membrane potential (*v*) and the phase angle (θ) based on the input current (*I*) and model parameter (τ).
  - Update rule: The *ThetaNeuron* model involves trigonometric operations to update θ and calculate the membrane potential (*v*).
  - Computational complexity: The *ThetaNeuron* model involves two multiplications and one addition for updating the membrane potential, and one addition for the theta phase update. It requires 3, 490 operations to update the neuron state in one-time step. The main operations involved are one multiplication and two additions for the theta frequency oscillator.
8. ***Izhikevich*** **model**
  - Mathematical description: The *IZH* model is a simplified neuron model that aims to capture a wide range of spiking behaviors with only two ordinary differential equations. It updates the membrane potential (*v*) and a recovery variable (*u*) based on the input current (*I*) and model parameters (*a*, *b*, *c*, and *d*).
  - Update rule: $\frac{dv}{dt}$ = 0.04 * *v*^2^ + 5 * *v* + 140 − *u* + *I* and $\frac{du}{dt}$ = *a* * (*b* * *v* − *u*).
  - Computational complexity: The *IZH* model involves two multiplications, two additions, and two subtractions in each update. It requires 6, 094 operations to update the neuron state in one-time step.
9. ***SRM*** **model**
  - Mathematical description: The *SRM* model incorporates synaptic interactions, and it updates the membrane potential (*v*) and synaptic variables (*s*, *r*) based on the input current (*I*), synaptic time constants (*tau*_*s*_, *tau*_*r*_), and synaptic weights.
  - Update rule: The *SRM* model involves three differential equations for $\frac{ds}{dt}$, $\frac{dr}{dt}$, and $\frac{dv}{dt}$.
  - Computational complexity: The *SRM* model involves three additions and two divisions for the spike response dynamics. It requires 4, 027 operations to update the neuron state in one-time step.

It is important to note that the provided explanation focuses on the mathematical descriptions and the number of operations in each update used in this study. The actual computational complexity of a machine learning model will depend on a wide range of factors, including the size of the data set, the complexity of the model architecture, the optimization algorithm used, and the hardware and software used to run the code. Therefore, the estimates provided by this study should be taken as rough approximations rather than precise measurements. As each models have additional parameters and details that can impact their overall behavior and applicability to specific neural simulations. However, these measurements still provide some insight into the relative computational costs of different models, which can be helpful for choosing between models or optimizing performance. Additionally, analyzing the computational complexity of a model can help identify potential bottlenecks or areas where optimization efforts may be most effective.

**Figure 3:**
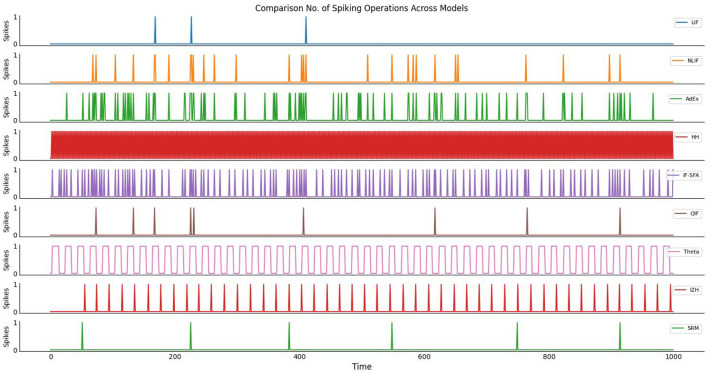
The plot illustrates the no. of spiking operations across different neuron models over time. Each neuron model represents a distinct mathematical description of how neurons function, and they vary in their computational complexity due to different update rules. The y-axis represents the number of spikes generated by each model, while the x-axis corresponds to the simulation time in arbitrary units.

Figure 3, visualize the comparison of a number of spiking operations across models. The number of operations required for a neuron model is an indication of how computationally expensive it is to simulate that model. In general, the more operations required, the longer it will take to simulate the behavior of that model. However, it's important to note that the actual number of operations required for a given model can vary depending on the specific implementation and hardware used to run the simulation. So while the results of your study suggest that the *HH* model is the most computationally expensive, this may not be true in all cases. Additionally, other factors such as memory usage and parallelization may also impact the overall computational complexity of a model.

#### 3.3.4. Biological plausibility

Comparing the biological plausibility of different neuron models can be a complex task, as each model makes different assumptions and simplifications about the underlying biology. However, some models may be considered more biologically plausible than others, depending on their ability to capture certain physiological phenomena. For example, the *HH* model is based on the biophysical properties of ion channels and can accurately reproduce the action potential firing of real neurons. The *AdEx* model also incorporates biophysical mechanisms such as adaptation and spike-frequency adaptation (SFA) but includes simplifications such as a single compartment and a fixed threshold. The *QIF* model is based on a simple integrate-and-fire mechanism, while the *NLIF* model includes a non-linear firing-rate function. The *LIF* model is one of the simplest and most widely used models, but it ignores many biophysical details of real neurons. The *ThetaNeuron* model is a modification of the *LIF* model that includes a theta-frequency oscillation.

**Figure 4:**
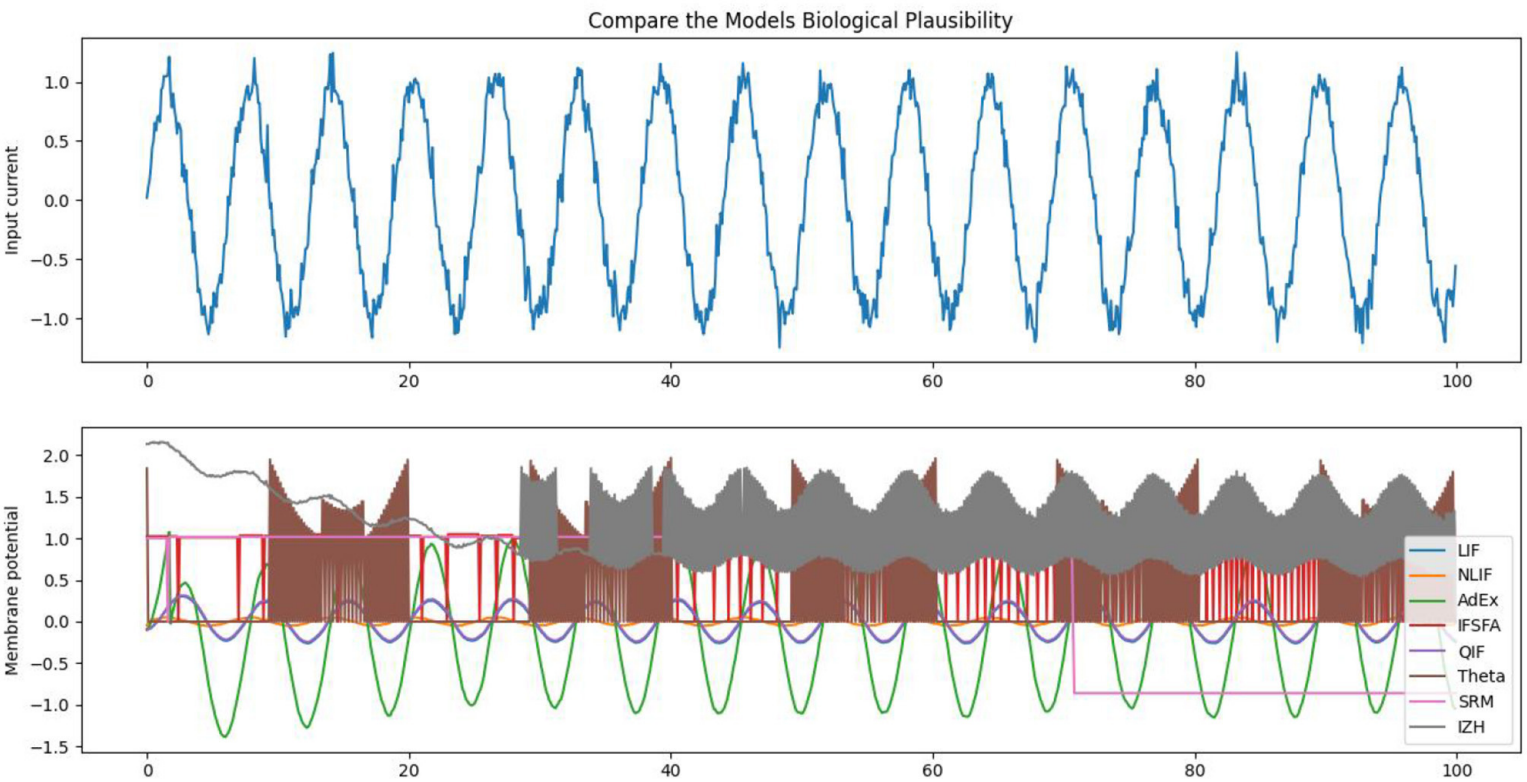
The figure illustrates the underlying biological plausibility of different neural models, each having specific parameters that influence the behavior of the neuron and highlighting how they can mimic the behavior of real neurons in response to input stimuli.

Each model in our study has its own set of parameters that determine how the neuron behaves. For example, the *LIF* model has parameters for the membrane time constant, reset voltage, firing threshold, initial voltage, and number of neurons. Some of the important parameter values can be seen in Table 3. The *NLIF* model has the same parameters as *LIF*, plus two additional parameters for the refractory period. The *AdEx* model has parameters for the membrane time constant, rheobase voltage, spike voltage, adaptation parameter, reset voltage, initial voltage, and the number of neurons. The *HH* model has parameters for the initial voltage and conductances for sodium, potassium, and leak channels. Each model also has a method called update, which takes as input the current input to the neuron and the time step, and returns a boolean value indicating whether the neuron has spiked in response to the input. Finally, each model has a weight matrix that determines the strength of the connections between neurons. The weight matrix is initialized randomly, with values drawn from a normal distribution with a mean of 0 and a standard deviation of 1. This random initialization ensures that the connections between neurons are not biased from the start. Figure 4, illustrates the biological plausibility of each model. This analysis serves as a crucial foundation for the subsequent exploration and understanding of these neuronal models in the study. The detailed results and code are available in our GitHub channel.

**Table 3:** Utilized parameter values for biological plausibility.

**Parameters**	**Parameter values used in biological plausibility**								
	**LIF**	**NLIF**	**AdEX**	**HH**	**IFSFA**	**QIF**	**Th.Nu**.	**SRM**	**IZH**
Time step	0.1	0.1	0.1	0.1	0.1	0.1	0.1	0.1	0.1
Tau (τ)	4.0	4.0	4.0	4.0	4.0	4.0	4.0	0.3	X
Reset potential (*V*_*reset*_)	0.0	0.0	0.1	–65	0.0	0.0	0.0	0.0	X
Threshold value (*V*_*th*_)	1.0	1.0	X	X	1.0	1.0	1.0	1.0	0.8
Initial potential (*V*_*init*_)	0.1	0.5	0.1	–65	–0.1	–0.1	0.0	0.0	0.01
Alpha (α)	X	0.5	X	X	X	X	X	X	X
Beta (β)	X	0.5	X	X	X	0.5	X	X	X
Rheobase potential (*V*_*rheo*_)	X	X	0.5	X	X	X	X	X	X
Threshold potential (*V*_*spike*_)	X	X	1.0	X	X	X	X	X	X
Slope factor (Δ_*T*_)	X	X	1.0	X	2.0	X	X	X	X
Activation variable for potassium (*n*)	X	X	X	0.3177	X	X	X	X	X
Activation variable for sodium (*m*)	X	X	X	0.0529	X	X	X	X	X
Inactivation variable for sodium (*h*)	X	X	X	0.5961	X	X	X	X	X
Adaptation time constant (τ_*w*_)	X	X	X	X	100	X	X	X	X
Adaptation conductance (*a*)	X	X	X	X	0.1	X	X	X	X
Adaptation control (*b*)	X	X	X	X	0.01	X	X	X	X
Synaptic time constants (τ_*r*_)	X	X	X	X	X	X	X	10	X
Recovery variable [RV] (*U*_*init*_)	X	X	X	X	X	X	X	X	0.2
Time scale RV (*a*)	X	X	X	X	X	X	X	X	0.02
Sensitivity RV (*b*)	X	X	X	X	X	X	X	X	0.2
After-spike reset MP (*c*)	X	X	X	X	X	X	X	X	0.1
After-spike reset RV (*d*)	X	X	X	X	X	X	X	X	0.06
Maximum conductances *g*_*Na*_	X	X	X	120	X	X	X	X	X
Maximum conductances *g*_*K*_	X	X	X	36	X	X	X	X	X
Maximum conductances *g*_*L*_	X	X	X	0.3	X	X	X	X	X
Sodium rev. pot. (*E*_*Na*_)	X	X	X	50	X	X	X	X	X
Potassium rev. pot. (*E*_*K*_)	X	X	X	–77	X	X	X	X	X
Leak conductance rev. pot. (*E*_*L*_)	X	X	X	–54.4	X	X	X	X	X
No. of neurons (*N*_*neurons*_)	1,000	1,000	1,000	1,000	1,000	1,000	1,000	1,000	1,000

#### 3.3.5. Future directions and limitations

This study contributes valuable insights into the behavior and performance of SNNs and their mathematical models. However, there are several future directions and limitations that should be addressed in further research:

**Future directions:**

- While this study focused on simulation comparisons of SNN models, future research should extend the investigation to hardware implementations. Testing these models on neuromorphic hardware or specialized hardware accelerators can provide a more realistic evaluation of their performance and energy efficiency in real-world applications.
- Evaluating the robustness and generalization capabilities of SNN models is essential for practical deployment. Future research should investigate how these models perform on more diverse datasets and under noisy input conditions to ensure their reliability in real-world scenarios.
- Investigating different spike encoding strategies can optimize information representation in SNNs. Research on efficient spike coding schemes can lead to enhanced performance and reduced computational overhead.
- Exploring various learning rules and synaptic plasticity mechanisms within SNNs can facilitate adaptive learning and memory capabilities. Incorporating online learning algorithms can enable SNNs to continuously update their parameters based on new data.
- Combining SNNs with traditional deep learning models or other machine learning techniques could harness the strengths of both approaches, leading to more powerful and versatile neural network architectures.

**Limitations:**

- The study's comparison of SNN models was conducted using a specific dataset (synthetic dataset), and the generalization to other datasets may vary. Future studies should include a broader range of datasets to ensure the models' performance across different scenarios.
- While SNN models aim for biological plausibility, implementing highly realistic biological models in simulations can be computationally demanding. Striking a balance between biological realism and efficiency remains a challenge.
- The study may not have exhaustively explored all possible hyperparameter settings for each SNN model. Optimizing model hyperparameters could potentially improve the overall performance of certain models.
- The study focused on smaller-scale SNN models, and their performance may differ when applied to larger networks or deep architectures. Evaluating the scalability of the models is crucial for their application in complex tasks.
- The precision of spike timing in SNNs can significantly impact their performance. Further investigations into temporal coding and precise spike timing mechanisms can enhance the capabilities of SNNs.

However, the current study provides valuable insights into SNN models' performance, behavior, and spike generation, future research should explore hardware implementations, robustness, and spike encoding strategies. Additionally, investigating learning rules, the hybrid approaches, and addressing the limitations of dataset diversity, biological plausibility, model hyperparameters, scalability, and spike timing precision can provide the way for more advanced and practical SNN applications in various domains.

## 4. Conclusion

SNNs are a powerful tool for simulating the behavior of neurons and have the potential for diverse applications. The *LIF*, *AdEx*, *NLIF*, *IF* − *SFA*, *ThetaNeuron*, *IZH*, *SRM*, *HH*, and *QIF* models are popular mathematical models used for simulating the spiking behavior of neurons in SNNs. However, selecting the most suitable model for specific applications, especially for classification tasks, can be challenging due to the demand for high accuracy and low-performance loss. To address this issue, a study was conducted to compare the performance, behavior, and spike generation methodology of different SNN models using the same inputs and neurons. The findings of this study offer valuable insights for researchers and practitioners in the field, emphasizing the importance of comparing different models to determine the most effective one.

The findings of the study highlight the significance of comparing multiple SNN models to identify the most effective one for a given application and emphasize the importance of selecting the most suitable model based on its biological plausibility and computational efficiency. The manuscript contributes to the research field by providing a deeper understanding of SNNs and their mathematical models, and by presenting a systematic approach to evaluating and selecting the most appropriate SNN model for classification tasks. The study's results can inform future research on developing more efficient and accurate SNN models, advancing progress in the field of artificial neural networks and their applications in various fields such as robotics, biomedicine, and cognitive science.

Furthermore, additional research could investigate other SNN models and compare their performance using various benchmarks to identify the most suitable model for specific applications, potentially saving time and resources. As SNNs continue to gain popularity, more attention must be given to developing reliable and efficient models. Overall, this study highlights the potential benefits of SNNs and their models and provides valuable insights for future research and development in this area.

## Author contributions

Sanaullah designed and conducted all experiments, evaluated the resulting data, and code written. Manuscript was primarily written by Sanaullah with contributions from SK. UR and TJ supervised the project and helped enhance and refine the manuscript. All authors contributed to the article and approved the submitted version.
